# An evaluation of the performance in the UK Royal College of Anaesthetists primary examination by UK medical school and gender

**DOI:** 10.1186/1472-6920-9-38

**Published:** 2009-06-29

**Authors:** Andrew R Bowhay, Simon D Watmough

**Affiliations:** 1 Centre for Excellence in Teaching and Learning, School Of Medical Education, The University of Liverpool, Cedar House, Ashton Street, Liverpool, L69 3GE, UK

## Abstract

**Background:**

There has been comparatively little consideration of the impact that the changes to undergraduate curricula might have on postgraduate academic performance. This study compares the performance of graduates by UK medical school and gender in the Multiple Choice Question (MCQ) section of the first part of the Fellowship of the Royal College of Anaesthetists (FRCA) examination.

**Methods:**

Data from each sitting of the MCQ section of the primary FRCA examination from June 1999 to May 2008 were analysed for performance by medical school and gender.

**Results:**

There were 4983 attempts at the MCQ part of the examination by 3303 graduates from the 19 United Kingdom medical schools. Using the standardised overall mark minus the pass mark graduates from five medical schools performed significantly better than the mean for the group and five schools performed significantly worse than the mean for the group. Males performed significantly better than females in all aspects of the MCQ – physiology, mean difference = 3.0% (95% CI 2.3, 3.7), p < 0.001; pharmacology, mean difference = 1.7% (95% CI 1.0, 2.3), p < 0.001; physics with clinical measurement, mean difference = 3.5% (95% CI 2.8, 4.1), p < 0.001; overall mark, mean difference = 2.7% (95% CI 2.1, 3.3), p < 0.001; and standardised overall mark minus the pass mark, mean difference = 2.5% (95% CI 1.9, 3.1), p < 0.001. Graduates from three medical schools that have undergone the change from Traditional to Problem Based Learning curricula did not show any change in performance in any aspects of the MCQ pre and post curriculum change.

**Conclusion:**

Graduates from each of the medical schools in the UK do show differences in performance in the MCQ section of the primary FRCA, but significant curriculum change does not lead to deterioration in post graduate examination performance. Whilst females now outnumber males taking the MCQ, they are not performing as well as the males.

## Background

Undergraduate education has undergone radical reform at most United Kingdom (UK) medical schools in the last two decades[1,2]. Undergraduate and postgraduate medical education are now viewed as a continuum in the training of a specialist consultant or General Practitioner (GP), yet there has been comparatively little consideration of the impact which changes in style of the learning curriculum content and objectives of undergraduate education might have on postgraduate performance[3,4]. Whilst some studies have focused on comparing competencies of graduates from traditional and Problem Based learning (PBL) curricula in the first postgraduate year[3,5,6], others have examined whether it is possible to predict the career path a graduate is likely to take from a particular medical school [7] or compared the perceived preparedness for the first postgraduate year across all UK universities[8]. However, there have been few studies comparing the outcomes of different medical curricula beyond the first year after graduation (Foundation 1 (F1) year). A small study in 1993 showed the pass rate per medical school for the Royal College of General Practitioners examination[9] and a more recent publication[4] for the examinations of the Royal College of Physicians have shown differences between the pass rates of graduates from UK medical schools.

The aims of this study were to compare the performance of graduates of UK medical schools in the postgraduate examinations in anaesthesia, which is the largest hospital based speciality in UK medicine. Performance in the written sections of the first part of the Fellowship of the Royal College of Anaesthetists (FRCA) examination were analysed in relation to undergraduate medical school as well as difference according to gender and type of undergraduate curriculum.

## Methods

The written part of the primary FRCA examination consists of a multiple choice question (MCQ) paper, 90 questions each with five parts (true/false format) lasting three hours, equally divided between pharmacology, physiology and biochemistry, physics and clinical measurement. The MCQ uses a negative marking system such that candidates are awarded one mark for a correct answer, minus one mark for an incorrect answer and no marks if the question is not answered. If candidates are successful in the written section of this examination they progress to an oral section which examines the same clinical subjects in more depth as well as resuscitation, anatomy, history-taking, physical examination and communication skills. The final part of the FRCA, which is generally taken 2 years after the primary, assesses clinical aspects of anaesthesia and intensive care medicine.

Ethical approval for this study was sought and gained by the Royal College of Anaesthetists (RCA) and the University of Liverpool School of Medical Education Research Ethics Committee. The data for analysis were collated from RCA spreadsheets for each sitting of the primary FRCA examination from June 1999 to May 2008 and from the main trainee database of the RCA, using the RCA registration number as the unique identifier. All data were anonymous. Only those candidates who had qualified from one of the 19 UK universities who were awarding medical degrees during this period were included. Only the university awarding the degree was recorded; as the medical schools in London are awarded a University of London degree all are recorded as "London".

Data available for analysis included the percentage mark for each section of the examination for each candidate as well as their overall final percentage mark, their closed mark for each part of the examination, RCA reference number, year and university of primary medical degree, date of birth and gender. Up to June 2007 the pass mark for each sitting of the MCQ was determined by norm-referencing; it was set 1% below the mean score for that sitting but dependent on the cohort ability. The ability of the cohort was determined by the discriminator ratio (DR): the ratio of the sum of the scores on discriminator questions in the current examination to the sum of the scores on the discriminator questions the last time that discriminator was used. The DR should be between 0.95 and 1.05 and if it was outside these limits then the cohort was considered to be significantly less or more able than average respectively. When a significant difference existed, an adjustment was made to the banding for the pass mark by placing it either closer to or further from the mean. The pass marks ranged from 46.4% to 59.4%. Since June 2007 this system has been complemented by Angoff Referencing which has made little difference to the pass marks. To aid the standardisation of results over the timescale of the data the difference between each candidate's overall mark and the pass mark for that sitting of the MCQ was determined. A candidate whose overall mark equalled the pass mark would therefore have a score of zero and a positive or negative score indicated the degree to which the candidate had passed or failed the examination.

### Statistical analysis

Data were analysed using SPSS 16.0. Comparison between groups was undertaken with independent sample t-tests or non parametric tests where appropriate, with p < 0.05 being considered significant. Data is presented as mean values with 95% confidence intervals unless otherwise stated.

## Results

From the June 1999 to the May 2008 sittings of the primary FRCA, there were 4983 attempts at the MCQ part of the examination by 3303 graduates from the 19 United Kingdom medical schools. Of the 4983 attempts at the primary MCQ, 1080 (21.7%) were poor fails and 3903 (78.3%) were able to progress to the oral section of the examination. There were 4056 attempts at the oral section of the examination with 2793 (68.9%) passing the examination (there is a discrepancy between this number of attempts and the 3903 who progressed from the MCQ; since June 2007 the MCQ has became a separate examination and candidates can make multiple attempts at the oral section without retaking the MCQ). The overall pass rate the examination was 56.1%. The median year of qualification was 1999 (range 1959 to 2006) with the median time from qualification to sitting the MCQ being 4.6 years (interquartile range 3.8 – 5.7). Overall 44.8% (medical school range 34 to 56%) of the trainees sitting the examination were female (see Additional file 1).

### Medical school effects

**Figure 1:**
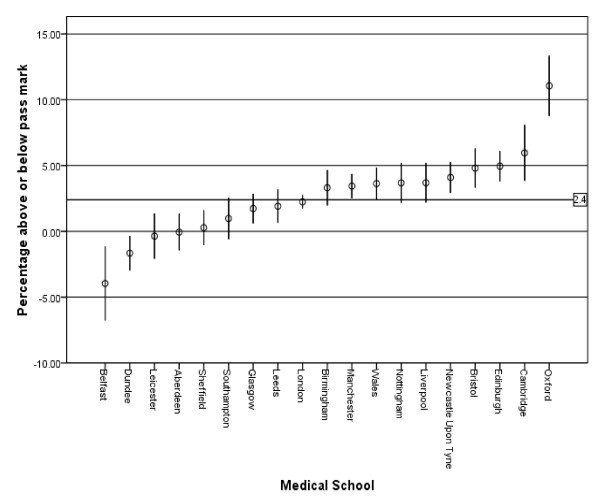
**Medical school effects for the MCQ section of the primary FRCA**. Medical schools are sorted by the size of the effect. Vertical bars show 95% confidence interval of the mean. Mean value for all medical schools = 2.4.

The performance of each medical school for each section of the MCQ, including the overall final mark and the standardised percentage mark above or below the pass mark is shown in Additional file 1. The effect of medical school on the percentage mark above or below the pass mark is shown graphically in figure 1. Five medical schools performed significantly better than the mean for the group – Oxford, Cambridge, Edinburgh, Bristol and Newcastle-upon-Tyne. Five schools performed significantly worse than the mean for the group – Sheffield, Aberdeen, Leicester, Dundee and Belfast. The likelihood of passing the primary FRCA examination at the first attempt for graduates of each medical school is shown in Table 1, with a mean first time pass rate of 62.2% (95% CI 60.5, 63.8) for the whole group (range 22.2% to 92.6%). Five medical schools had first time pass rates significantly better than the mean for the group – Oxford, Cambridge, Nottingham, Birmingham and Edinburgh; two medical schools first time pass rates were significantly worse than the mean for the group – Belfast and Dundee.

**Table 1:** Number of graduates of each Medical School, as well as males and females, completing the primary FRCA examination and those that passed the examination first time and with the first time pass rates, with lower and upper 95% confidence intervals

Medical School	Number Completing Exam	Number Passing First Time	First Time Pass rate (%) with 95% Confidence Intervals (lower limit, upper limit)
Oxford	54	50	92.6 (83.3, 97.5)
Cambridge	64	52	81.3 (70.4, 89.3)
Nottingham	151	112	74.2 (66.8, 80.7)
Birmingham	156	114	73.1 (65.7, 79.6)
Edinburgh	151	109	72.2 (64.7, 78.9)
Newcastle Upon Tyne	111	77	69.4 (60.4, 77.4)
Bristol	139	96	69.1 (61.1, 76.3)
Wales	121	80	66.1 (57.4, 74.1)
Manchester	243	158	65.0 (58.9, 70.8)
Liverpool	111	69	62.2 (52.9, 70.8)
Leeds	118	73	61.9 (52.9, 70.3)
Southampton	104	63	60.6 (51.0, 69.6)
London	949	569	60.0 (56.8, 63.0)
Sheffield	141	81	57.5 (49.2, 65.4)
Leicester	98	54	55.1 (45.2, 64.7)
Glasgow	194	105	54.1 (47.1, 61.0)
Aberdeen	127	67	52.8 (44.1, 61.3)
Dundee	107	40	37.4 (28.7, 46.8)
Belfast	45	10	22.2 (12.0, 35.9)
Male	1774	1133	63.9 (61.6, 66.1)*
Female	1410	846	60.0 (57.4, 62.5)*
All	3184	1979	62.2 (60.5, 63.8)

* Chi squared = 4.99, p = 0.025

Medical schools listed in rank order of the pass rate.

Medical school curricula are constantly changing, and it has not been possible in this study to look for an effect of changes in balance of the Basic Science Curriculum. We did however look at the results in three schools where the curriculum changed to PBL. We selected this curriculum change not out of any belief that it is likely to be relevant but because this aspect of curriculum design has previously attracted attention [3,5,6]. Three medical schools have undertaken the change from traditional curricula to PBL curricula; Manchester in 1994, Liverpool and Glasgow both in 1996. Assuming that each course takes 5 years then the first graduates from these new curricula were awarded their degrees in 1999 and 2001 respectively. Graduates of these medical schools pre and post curricula change did not show any change in performance in any aspects of the MCQ. For the mean standardised percentage above and below pass mark (%): Manchester graduates pre and post 1999 = 4.1 (95% CI 2.9, 5.4) vs 2.8 (95% CI 1.4, 4.2), p = 0.17; Liverpool graduates pre and post 2001 = 3.5 (95% CI 0.03, 6.9) vs 3.8 (95% CI 2.3, 5.3), p = 0.86; Glasgow graduates pre and post 2001 = 2.2 (95% CI 0.9, 3.4) vs 0.6 (95%CI -1.8, 3.1), p = 0.23.

### Gender effects

**Figure 2:**
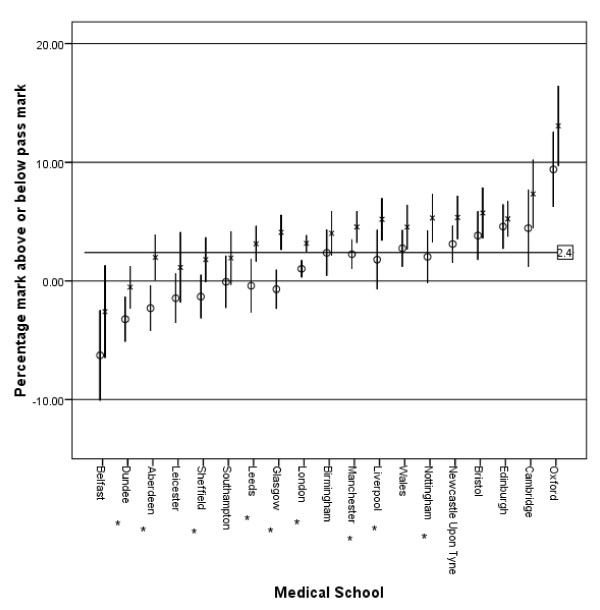
**Gender and medical school effects for the MCQ section of the primary FRCA**. Medical schools are sorted by the size of the effect. Vertical bars show 95% confidence interval of the mean. x = Male, o = Female. Mean value for both genders = 2.4. * p ≤ 0.05 for difference between the genders.

**Figure 3:**
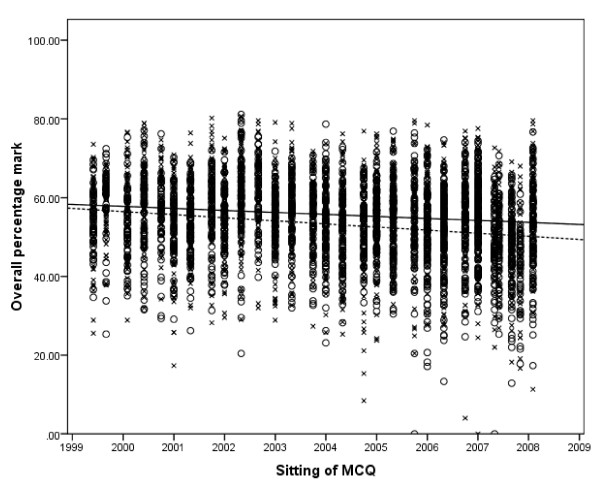
**Scatter graph of overall performance for both genders for each sitting of the MCQ with linear trend lines for each gender**. Markers: o = female, x = male. Trend lines: --- = female, -- = male.

Males performed significantly better in all aspects of the MCQ. For physiology, mean difference = 3.0% (95% CI 2.3, 3.7), p < 0.001; pharmacology, mean difference = 1.7% (95% CI 1.0, 2.3), p < 0.001; physics with clinical measurement, mean difference = 3.5% (95% CI 2.8, 4.1), p < 0.001; overall mark, mean difference = 2.7% (95% CI 2.1, 3.3), p < 0.001; and standardised overall mark minus the pass mark, mean difference = 2.5% (95% CI 1.9, 3.1), p < 0.001. The overall pass rate for the examination was 53.5% for females and 58.2% for males (Chi squared = 11.2, p = 0.001). The variation in gender performance by medical school is shown in figure 2 with the difference in performance by males being significant in nine medical schools. The performance in the three parts of the MCQ, and hence overall performance, declined for both sexes from 1999 to 2008, with females showing a greater decline than males (figure 3). Overall females made 2288 attempts at the MCQ with 1710 (74.7%) progressing to the oral section of the examination; this is compared to 2193 of 2695 (81.4%) attempts for males (Chi squared = 31.7, p < 0.001). Of those female candidates who undertook the oral section of the examination 1224 of 1837 (66.6%) attempts passed; for males, 1569 of 2219 (70.7%) attempts led to a pass (Chi squared = 7.6, p = 0.006). The likelihood of each gender passing the primary examination at the first attempt is shown in Table 1, with the first time pass rate for males being 63.9% (95% CI 61.6, 66.1) compared to 60.0% (95% CI 57.4, 62.5) for females (Chi squared = 4.99, p = 0.025).

### Correlations between parts of the MCQ

There was a highly significant correlation between the 3 parts of the MCQ; physiology with pharmacology, r = 0.69, n = 4893; pharmacology with physics and clinical measurement, r = 0.65, n = 4893; physiology and clinical measurement, r = 0.63, n = 4893.

## Discussion

The results do show that there are differences between medical schools in the UK on graduate performance in the FRCA examination. A graduate from Oxford, Cambridge, Birmingham, Nottingham and Edinburgh is more likely to pass the primary FRCA at the first attempt than a graduate from Belfast or Dundee. These results are in some agreement with the medical school graduate performance in the MRCP examination[4] with Oxford, Cambridge, Edinburgh and Newcastle upon Tyne being in the top quartile and Belfast, Dundee, Aberdeen in the bottom quartile of both series.

A study at the University of Liverpool has shown that 5–6 years after graduation, trainees do feel their undergraduate education does impact on their competencies as doctors[10,11] and are able to relate their experiences to their undergraduate education, suggesting that undergraduate education has some impact on post graduate performance. In addition there is some correlation with these results and the rankings in the 2008 *Times *survey grading medical schools[12] with the top quartile medical schools (Oxford, Cambridge, Edinburgh, Bristol and Newcastle) being ranked 1,2,3 = 9^th ^and 16^th ^respectively and the bottom quartile medical schools (Sheffield, Aberdeen, Leicester, Dundee and Belfast) being ranked 19^th^, 5^th^, 18^th^, 22^nd ^and 20^th ^respectively. However, the performance in post graduate examination does not correlate with career choice as those medical school graduates who are most likely to pursue a career in anaesthesia are from Bristol, Edinburgh and Southampton whilst Oxford and Cambridge graduates are less likely to make that career choice[13].

Female graduates are underperforming in both the MCQ and oral parts of the primary FRCA examination compared to male graduates, and are less likely to pass the examination at the first attempt. The underperformance by females has also been found in Parts 1 and 2 of the Membership of the Royal College of Physicians examination (MRCP(UK))[4], both of which are MCQ type examinations, however they performed better than males in the clinical assessment section (PACES) of the examination[14]. This is in contrast to performance at medical school where females tend to outperform males[15,16] and are more likely to be awarded an honours degree[17]. In school science examinations male students have historically outperformed female school students although this has recently been reversed[18]. One reason suggested for this change is that the assessment system in schools now favours female students compared to male students[19]. The revision strategy of male students of 'cramming-it-all-in-at-the-last-minute' is perhaps less beneficial for assessment throughout the year for modular examinations and coursework, but the "cramming" approach may be best suited for final summative assessments such as postgraduate medical examinations. In 1974 females comprised approximately 27% of those qualifying from medical school[13], but by 1991 50% of the medical student population were female and by 2005 constituted 61% of the undergraduate student population[20]. So after years of underrepresentation, females now outnumber males[21] in the medical workforce in the UK: Although overall 44.8% of candidates were female in this study their representation increased for each year of qualification, such that for those qualifying after 2002 58% were female; this is similar to the 58% of doctors who were female who responded to a BMA survey[22] in 2006. The primary FRCA examination format has not changed during the period of the study, however other factors may affect gender performance such as part time training, which may lead to difficulties with career progression[23] although this has been disputed[24], and the stresses involved in achieving a satisfactory work-life balance[20]. However, there are concerns that negative marking, which is intended to correct for guessing, may discriminate between students on their risk taking behaviour[25] and as female students are less likely to take risks[26] this could lead to gender bias. The RCA is aware of these concerns and negative marking has been replaced by number-right scoring in all its examinations from September 2008.

The Universities of Manchester, Glasgow and Liverpool introduced integrated problem-based learning curricula replacing their traditional lecture based courses in 1994 and 1996 respectively. There have been concerns expressed about these recent reforms [27] in UK medical education and fears that moving to a PBL system may have a negative impact on the basic science knowledge of PBL graduates which may impact on the ability of PBL to pass science based postgraduate exams[28,29]. These results suggest that reforming a medical curriculum from a traditional course to an integrated PBL in the UK does not impact on the ability of graduates to undertake science based post graduate examinations; this is confirmed by results from studies in North America[30-32] which have also shown there are no significant differences between PBL and traditional graduates on their licensing exams. Although there is variation in the content of medical school curricula in the UK, all medical schools have to incorporate the recommendations of *Tomorrow's Doctors*[33]into their curricula but this data confirms that some elements of performance do not change.

There was a highly significant correlation between the 3 parts of the MCQ. It would be expected that all candidates would have prior knowledge of physiology and pharmacology, but it is unlikely that any of the candidates will have been taught and examined on physics with clinical measurement. This therefore tests their ability to attain and apply new knowledge and it would be expected that students who do well in this area would also be better able to apply previous physiology or pharmacology knowledge from their undergraduate education.

There are some limitations to the study. Only a small percentage of medical graduates will take the anaesthesia examinations, therefore performance in these examinations cannot be extrapolated to all post graduate examinations. Although the recent introduction of Modernising Medical Careers (MMC) for postgraduate medical education has resulted in some reform of medical training in the UK, this will have had little impact on the results of this study as the pre and post MMC trainees have experienced similar training pathways. However, the exposure of undergraduates to anaesthesia will vary between medical schools; for instance, with the advent of student selected components into undergraduate curricula students can voluntarily undertake an undergraduate attachment in anaesthesia. In addition MMC has allowed foundation doctors the opportunity to take a short rotation in anaesthesia, or a related specialty such as intensive care medicine; exposure that would more likely lead to a career in the speciality, but would not necessarily lead to an improvement in overall results. A confounding factor may be that even if graduates remain close to their medical school where they qualified the differences in performance may rather reflect the quality of postgraduate course available for anaesthesia trainees in that locality.

## Conclusion

These results show that graduates from each of the medical schools in the UK do have differences in performance in the MCQ part of the primary FRCA. Whilst females now outnumber males taking the examination, it is concerning that females do not seem to be performing as well as males.

## Abbreviations

BMA: British Medical Association; CI: Confidence Interval; FRCA: Fellowship of the Royal College of Anaesthetists; GMC: General Medical Council; GP: General Practitioner; MCQ: Multiple Choice Questions; MMC: Modernising Medical Careers; MRCP: Membership of the Royal College of Physicians; OSCE: Objectively Structured Clinical Examination; PBL: Problem Based learning; RCA: Royal College of Anaesthetists; SOE: Structured oral Examination; UK: United kingdom.

## Competing interests

The authors declare that they have no competing interests.

## Authors' contributions

AB conceived and designed the study, analysed and interpreted the data, drafted the article and approved the final version to be published. SW conceived and designed the study, drafted the article and approved the final version to be published.

## Pre-publication history

The pre-publication history for this paper can be accessed here:



## Supplementary Material

Additional file 1 **Table of MCQ results by medical School**. Table showing mean values, with lower and upper 95% confidence intervals, for each section of the MCQ by Medical School and number of graduates entering the Primary FRCA examination by medical school and gender. Click here for file
