# Supplementary material for: An evaluation of the performance in the UK Royal College of Anaesthetists primary examination by UK medical school and gender
Source: BMC Med Educ. 2009 Jun 29;9:38. doi: 10.1186/1472-6920-9-38 (PMC2707380; doi:10.1186/1472-6920-9-38)
Supplement: Additional file 1 — Table of MCQ results by medical School. Table showing mean values, with lower and upper 95% confidence intervals, for each section of the MCQ by Medical School and number of graduates entering the Primary FRCA examination by medical school and gender. [file 1472-6920-9-38-S1.doc]

Mean values with lower and upper 95% confidence intervals for each section of the MCQ by Medical School and number of graduates entering the Primary FRCA examination by medical school and gender.

|  | Pharmacology | Physiology | Physics & Clinical Measurement | Overall Result | Overall Result-Pass Mark |  |  |  |
| --- | --- | --- | --- | --- | --- | --- | --- | --- |
| Medical School | Mean (%) with 95% Confidence Intervals  (lower limit, upper limit) | Mean (%) with 95% Confidence Intervals  (lower limit, upper limit) | Mean (%) with 95% Confidence Intervals (lower limit, upper limit) | Mean (%) with 95% Confidence Intervals (lower limit, upper limit) | Mean (%) with 95% Confidence Intervals (lower limit, upper limit) | Number entering examination from each medical school | Female | Percent Female |
| Aberdeen | 50.9 (49.3, 52.5) | 53.7 (52.0, 55.3) | 51.5 (49.9, 53.1) | 52.0 (50.6, 53.5) | -0.1 (-1.4, 1.3) | 132 | 58 | 43.9% |
| Belfast | 47.3 (43.9, 50.7) | 48.7 (45.5, 51.9) | 47.1 (44.1, 50.2) | 47.7 (44.9, 50.6) | -4.0 (-6.8, -1.2) | 46 | 18 | 39.1% |
| Birmingham | 55.1 (53.5, 56.6) | 57.3 (55.7, 58.9) | 54.3 (52.6, 55.9) | 55.5 (54.1, 57.0) | 3.3 (2.0, 4.7) | 162 | 65 | 40.1% |
| Bristol | 55.4 (53.7, 57.2) | 58.2 (56.6, 59.9) | 57.1 (55.3, 58.8) | 56.9 (55.4, 58.4) | 4.8 (3.3, 6.3) | 141 | 68 | 48.2% |
| Cambridge | 56.0 (53.6, 58.4) | 59.2 (56.9, 61.4) | 57.4 (54.9, 59.8) | 57.5 (55.4, 59.6) | 6.0 (3.8, 8.1) | 68 | 33 | 48.5% |
| Dundee | 49.5 (47.9, 51.0) | 51.7 (50.2, 53.3) | 50.0 (48.4, 51.6) | 50.4 (49.0, 51.8) | -1.7 (-3.0, -0.3) | 113 | 47 | 41.6% |
| Edinburgh | 56.3 (54.8, 57.8) | 60.0 (58.6, 61.3) | 56.3 (54.9, 57.6) | 57.5 (56.3, 58.6) | 5.0 (3.8, 6.1) | 155 | 69 | 44.5% |
| Glasgow | 53.9 (52.6, 55.1) | 56.1 (54.8, 57.4) | 52.1 (50.6, 53.5) | 54.0 (52.8, 55.2) | 1.7 (0.6, 2.9) | 200 | 99 | 49.5% |
| Leeds | 53.0 (51.3, 54.6) | 55.8 (54.2, 57.4) | 54.0 (52.4, 55.5) | 54.3 (52.9, 55.6) | 1.9 (0.6, 3.2) | 123 | 42 | 34.1% |
| Leicester | 50.7 (48.6, 52.7) | 53.6 (51.6, 55.5) | 51.2 (49.1, 53.2) | 51.8 (50.0, 53.6) | -0.4 (-2.1, 1.4) | 100 | 56 | 56.0% |
| Liverpool | 53.9 (52.1, 55.6) | 56.5 (54.8, 58.2) | 56.6 (55.0, 58.2) | 55.7 (54.2, 57.1) | 4.0 (2.6, 5.4) | 116 | 49 | 42.2% |
| London | 53.5 (52.8, 54.1) | 56.1 (55.5, 56.7) | 53.7 (53.1, 54.3) | 54.4 (53.9, 54.9) | 2.2 (1.7, 2.8) | 984 | 408 | 41.5% |
| Manchester | 54.8 (53.8, 55.9) | 57.4 (56.2, 58.5) | 54.6 (53.6, 55.7) | 55.6 (54.7, 56.6) | 3.4 (2.5, 4.4) | 252 | 116 | 46.0% |
| Newcastle Upon Tyne | 55.3 (53.8, 56.8) | 58.5 (57.0, 60.0) | 55.1 (53.5, 56.6) | 56.3 (55.0, 57.5) | 4.1 (2.9, 5.3) | 121 | 67 | 55.4% |
| Nottingham | 54.7 (53.0, 56.4) | 56.9 (55.1, 58.6) | 54.6 (52.8, 56.4) | 55.4 (53.8, 57.0) | 3.7 (2.2, 5.2) | 158 | 82 | 51.9% |
| Oxford | 62.2 (59.8, 64.6) | 64.5 (61.5, 67.4) | 62.0 (59.3, 64.7) | 62.9 (60.6, 65.2) | 11.1 (8.8, 13.4) | 55 | 29 | 52.7% |
| Sheffield | 51.6 (50.1, 53.0) | 52.5 (50.8, 54.2) | 52.0 (50.5, 53.5) | 52.0 (50.7, 53.4) | 0.3 (-1.1, 1.6) | 146 | 67 | 45.9% |
| Southampton | 52.2 (50.4, 54.1) | 54.9 (53.0, 56.8) | 52.2 (50.5, 54.0) | 53.1 (51.5, 54.7) | 1.0 (-0.6, 2.6) | 106 | 46 | 43.4% |
| Wales | 55.1 (53.6, 56.7) | 57.2 (55.6, 58.9) | 55.0 (53.5, 56.5) | 55.8 (54.5, 57.1) | 3.6 (2.4, 4.8) | 125 | 61 | 48.8% |
| All | 53.6 (53.3, 53.9) | 56.1 (55.8, 56.4) | 53.8 (53.4, 54.1) | 54.5 (54.2, 54.8) | 2.4 (2.1, 2.7) | 3303 | 1480 | 44.8% |

Medical schools listed in alphabetical order.
